# Appendiceal Hydatid Disease Presenting as Acute Appendicitis: A Case Report and Review of the Literature

**DOI:** 10.7759/cureus.117833

**Published:** 2026-10-13

**Authors:** Samer Al Hakkak, Mahmood Albo Ahmed, Shahad Al-Mashhadani

**Affiliations:** 1 Surgery, College of Medicine, University of Alkafeel, Najaf, IRQ; 2 Gastroenterology, Mid Yorkshire Hospitals NHS Trust, Wakefield, GBR; 3 General Medicine, Baghdad Medical City, Baghdad, IRQ

**Keywords:** acute appendicitis, appendiceal hydatid disease, cystic echinococcosis, echinococcus granulosus, hydatid cyst, intraoperative rupture, parasitic appendicitis

## Abstract

Appendiceal hydatid disease is an exceptionally rare manifestation of cystic echinococcosis and may be indistinguishable from routine acute appendicitis before surgery. We report a 43-year-old man who presented with a two-day history of right lower-quadrant abdominal pain, anorexia, and nausea. Clinical examination demonstrated localized tenderness at McBurney’s point with signs of localized peritoneal irritation. Laboratory investigations showed leukocytosis and an elevated C-reactive protein level. Abdominal ultrasonography demonstrated a non-compressible, inflamed appendix measuring approximately 9 mm in diameter with a fecalith-like intraluminal structure, and emergency open appendectomy was undertaken for presumed acute appendicitis. Intraoperatively, an unexpected cystic lesion was identified adjacent to the appendiceal base and ruptured during dissection, with extrusion of whitish membrane-like material. The operative field was immediately irrigated with hypertonic saline, and no intraoperative anaphylactic reaction occurred. Histopathological examination demonstrated laminated and germinal layers consistent with cystic echinococcosis. The patient subsequently received albendazole 400 mg twice daily in three 28-day cycles separated by 14-day treatment-free intervals. Follow-up computed tomography performed three months after surgery demonstrated no residual or additional hydatid cysts at that time, and the patient remained clinically well without evidence of recurrence during 12 months of follow-up. This case, considered alongside the published literature, highlights the diagnostic difficulty of appendiceal hydatid disease, the importance of cautious handling of unexpected cystic appendiceal lesions, and the need for postoperative antiparasitic treatment and imaging surveillance.

## Introduction

Cystic echinococcosis (hydatid disease) is a zoonotic infection caused by the larval stage of *Echinococcus* species, most commonly *Echinococcus granulosus sensu lato*. Humans are accidental intermediate hosts after ingestion of parasite eggs. The liver and lungs are the predominant sites because they act as the principal vascular filters, although cysts may occur in almost any organ [1,2]. Following ingestion, the parasite penetrates the intestinal mucosa and typically reaches the liver through the portal circulation, with the lungs acting as a second major filter. Unusual extrahepatic sites may occur through alternative hematogenous or lymphatic dissemination [1,2]. Current World Health Organization (WHO) and Centers for Disease Control and Prevention (CDC) guidance emphasizes organ- and stage-specific management of cystic echinococcosis and careful avoidance of iatrogenic complications [3,4].

Appendiceal involvement is exceptionally uncommon. Published reports include hydatid elements within the appendiceal lumen, cysts arising from the appendiceal wall or mesoappendix, secondary appendiceal involvement in disseminated disease, and periappendiceal or right iliac fossa cysts mimicking appendiceal masses [5-12]. Clinical findings are non-specific and may resemble uncomplicated acute appendicitis, an appendiceal mucocele, an adnexal mass, or an inflammatory right iliac fossa mass. Consequently, the diagnosis is often made intraoperatively or by histopathology.

The major operative concern is rupture of an unrecognized cyst. Exposure to antigen-rich hydatid fluid may cause hypersensitivity or anaphylaxis, while viable protoscolices may disseminate throughout the peritoneum and produce secondary echinococcosis [13,14]. Serological testing may support the diagnosis but has variable sensitivity [15]. Histopathological examination may demonstrate characteristic laminated and germinal layers when the diagnosis is unsuspected [16]. We present a case of appendiceal hydatid disease discovered during emergency appendectomy and complicated by intraoperative rupture, followed by a case-based narrative review focused on presentation, diagnosis, operative management, antiparasitic treatment, and follow-up.

## Case presentation

A 43-year-old man presented to the emergency department with a two-day history of right lower-quadrant abdominal pain accompanied by anorexia and nausea. He had no known previous hepatic or pulmonary hydatid disease. Abdominal examination demonstrated localized tenderness at McBurney’s point with signs of localized peritoneal irritation. His admission vital signs and routine laboratory investigations are summarized in Table 1.

**Table 1:** Clinical timeline of the patient's presentation, management, and follow-up. Admission vital signs and routine laboratory investigations. Reference intervals may vary between laboratories; the ranges shown represent standard adult reference ranges and should be replaced with the reporting institution’s laboratory-specific ranges if available. mmHg: millimeters of mercury

Parameter	Patient result	Reference value/range
Blood pressure	91/57 mmHg	Approximately 90-120/60-80 mmHg
Heart rate	117 beats/min	60-100 beats/min
Respiratory rate	23 breaths/min	12-20 breaths/min
Oxygen saturation on room air	96%	≥95%
Temperature	37.9°C	36.0°C-37.5°C
Hemoglobin (Hb)	127 g/L	130-170 g/L
White cell count (WCC)	15.3 × 10⁹/L	4.0-11.0 × 10⁹/L
Platelet count	377 × 10⁹/L	150-400 × 10⁹/L
C-reactive protein (CRP)	92 mg/L	<5 mg/L
Creatinine	52 µmol/L	60-110 µmol/L
Urea	6.9 mmol/L	2.5-7.8 mmol/L
Sodium (Na)	143 mmol/L	135-145 mmol/L
Potassium (K)	4.1 mmol/L	3.5-5.0 mmol/L
Alanine aminotransferase (ALT)	34 U/L	0-40 U/L
Alkaline phosphatase (ALP)	110 U/L	30-130 U/L
Total bilirubin	9 µmol/L	0-21 µmol/L

Abdominal ultrasonography was reported as demonstrating a non-compressible, inflamed appendix measuring approximately 9 mm in diameter with a fecalith-like intraluminal structure. No hydatid cyst was recognized preoperatively. The original ultrasonographic images were not available to the authors at the time of manuscript preparation; therefore, the ultrasonographic findings reported here are derived from the contemporaneous radiology documentation.

Because the clinical and ultrasonographic findings supported a diagnosis of acute appendicitis, emergency open appendectomy was performed. The appendix was acutely inflamed. During mobilization and dissection near the appendiceal base, an unexpected cystic lesion closely related to the appendiceal stump was encountered (Figure 1). During manipulation, the lesion ruptured with leakage of cyst contents and extrusion of whitish membrane-like material (Figure 2). A wider operative view demonstrated the exteriorized membrane-like hydatid material following rupture (Figure 3).

**Figure 1:**
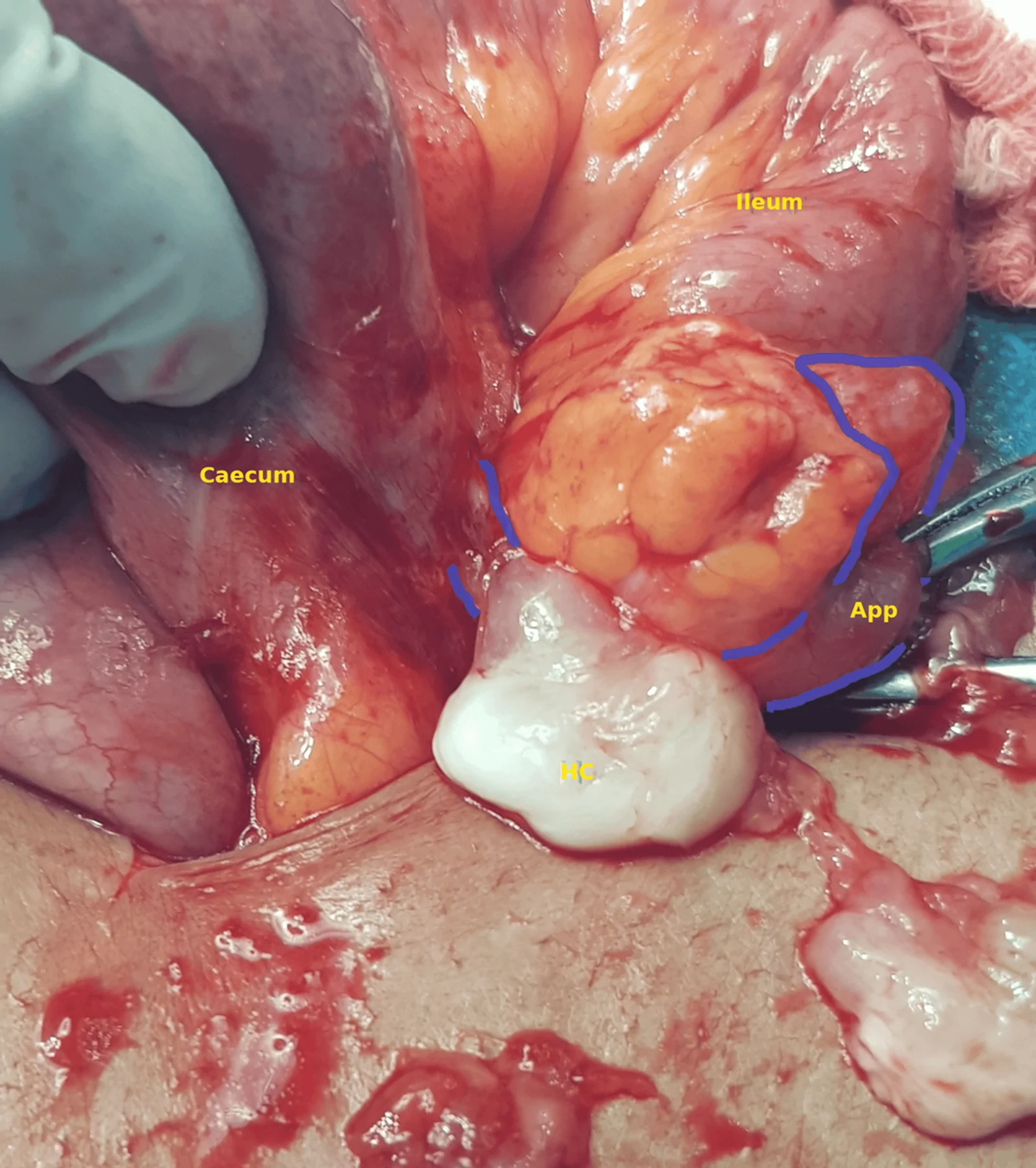
Intraoperative view demonstrating the primary appendiceal hydatid cyst (HC) adjacent to the appendiceal base. Intraoperative photograph demonstrating a cystic lesion arising adjacent to the appendiceal base before rupture. The cecum, terminal ileum, appendix (App), and HC are identified. The lesion appeared as an unexpected cystic mass during emergency appendectomy performed for presumed acute appendicitis.

**Figure 2:**
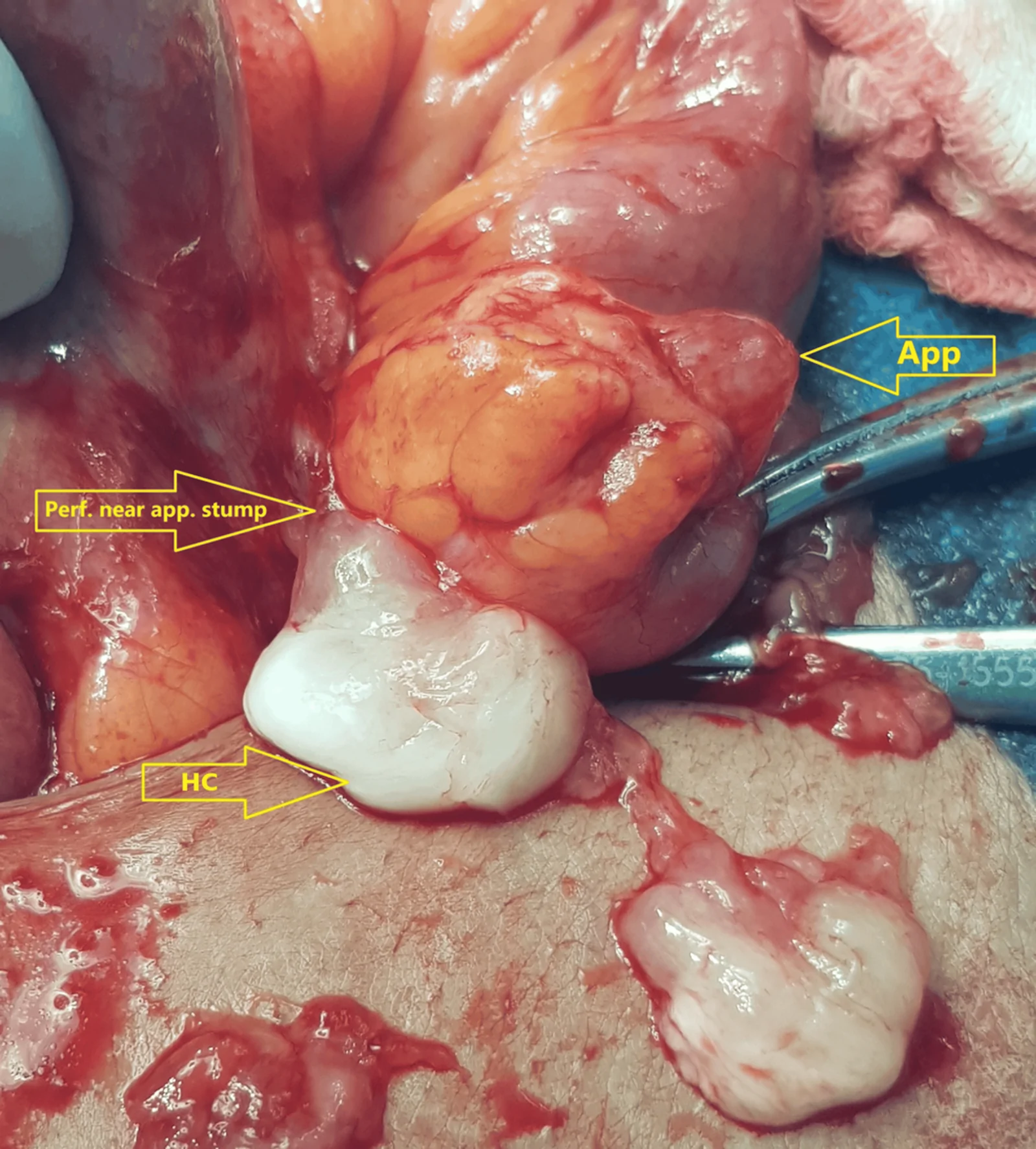
Intraoperative rupture of the appendiceal hydatid cyst (HC) with leakage of cyst contents. Annotated intraoperative photograph demonstrating the site of perforation adjacent to the appendiceal stump following accidental rupture of the HC. The appendiceal remnant and extruded HC are identified. Immediate operative precautions, including hypertonic saline irrigation, were undertaken to minimize the risk of peritoneal dissemination. App: appendix; Perf. near app. stump: perforation near the appendiceal stump

**Figure 3:**
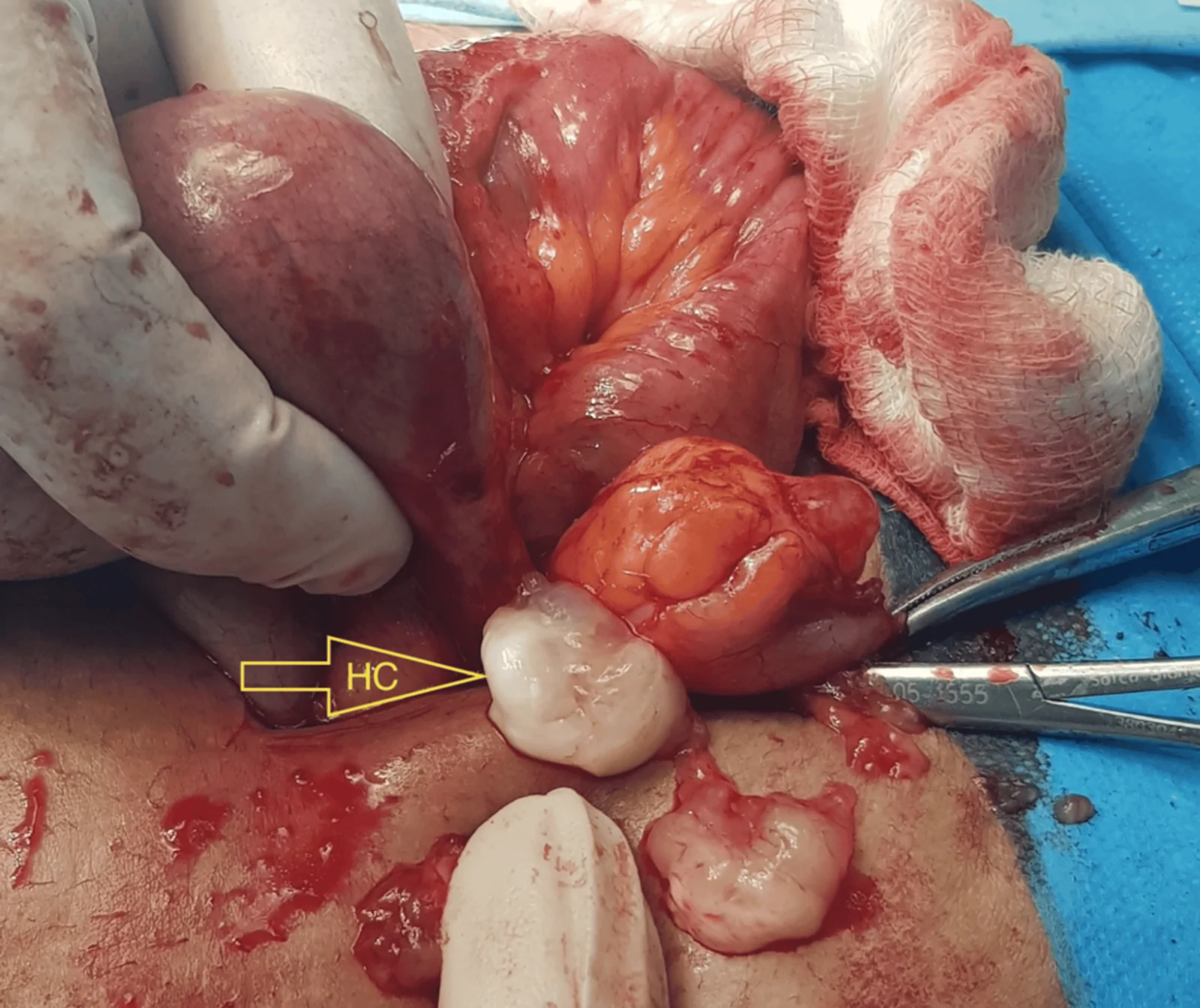
Wider operative view following rupture of the appendiceal hydatid cyst (HC). Wider intraoperative view demonstrating the ruptured appendiceal HC with exteriorized laminated membrane representing HC contents following accidental intraoperative rupture. The surrounding operative field after appendectomy is shown before completion of irrigation and definitive management.

The operative field was immediately controlled and irrigated with hypertonic saline to reduce the risk of peritoneal dissemination. Appendectomy was completed, and the specimen together with the membrane-like material was submitted for histopathological examination. No intraoperative anaphylactic reaction was documented.

Histopathological examination demonstrated a thick, pale eosinophilic, acellular laminated layer of the hydatid cyst wall, with adjacent host fibrous tissue and chronic inflammatory infiltrate. The contemporaneous histopathology report also documented laminated and germinal layers, confirming cystic echinococcosis and appendiceal hydatid disease (Figure 4).

**Figure 4:**
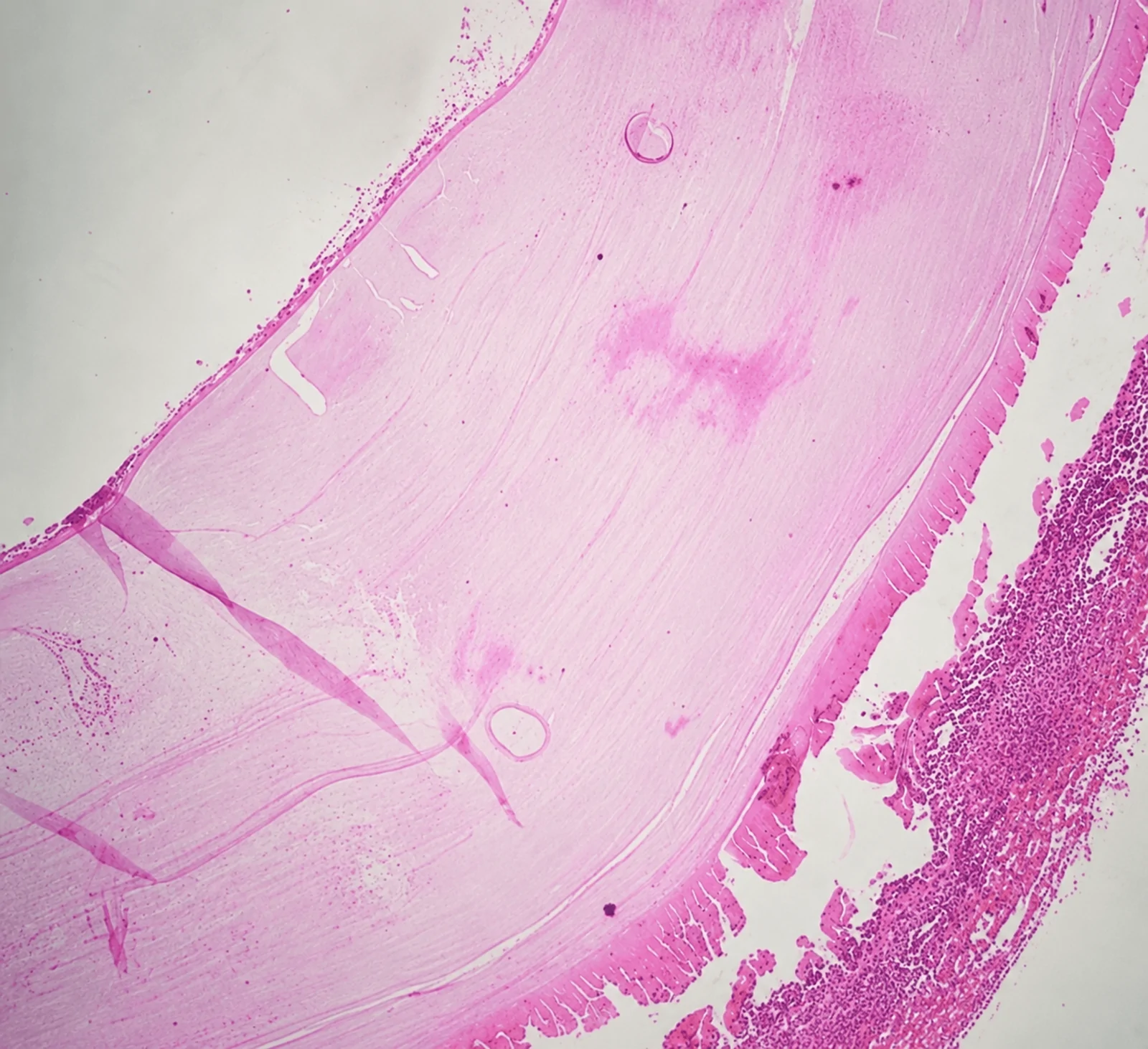
Histopathological appearance of the hydatid cyst wall. Low-power hematoxylin and eosin-stained section demonstrating the acellular laminated layer, germinal layer, and adjacent host fibrous and inflammatory tissue. These histopathological features are consistent with cystic echinococcosis.

Postoperatively, the patient was treated with albendazole 400 mg orally twice daily for 28 days per cycle. Three cycles were administered, with a 14-day treatment-free interval between cycles. A follow-up contrast-enhanced computed tomography (CECT) scan of the abdomen performed three months after surgery demonstrated no residual or additional hydatid cysts at that time. The patient was followed clinically for 12 months, with no evidence of recurrence during the follow-up period.

## Discussion

Review methods

A targeted narrative literature search was performed on August 5, 2026. PubMed/MEDLINE and publisher-indexed web records were searched using combinations of the terms “appendiceal hydatid”, “appendicular hydatid”, “hydatid cyst appendix”, “Echinococcus appendix”, and “hydatidosis appendicitis”. Reference lists of retrieved reports were also reviewed. Human reports describing hydatid disease arising from, involving, or closely mimicking disease of the appendix were eligible. Reports were included only when the bibliographic record and the relevant clinical details could be verified from PubMed, PubMed Central, the publisher page, or an indexed journal record. Reviews without a new case, animal studies, and reports without sufficient retrievable clinical information were excluded. The review was designed as a focused narrative synthesis rather than a systematic review or meta-analysis; reporting was informed by the CARE case-report framework and the Scale for the Assessment of Narrative Review Articles (SANRA) principles for narrative reviews [17,18].

Because the evidence consists almost entirely of isolated case reports, no pooled inferential analysis was attempted. Findings are presented descriptively, and distinctions are maintained between primary appendiceal disease, secondary appendiceal involvement, and periappendiceal lesions mimicking appendiceal pathology.

Findings from the literature review

The verified literature demonstrates marked heterogeneity. Some cases presented as classic acute appendicitis and were diagnosed only on histopathology [5,6], whereas others appeared as chronic pelvic or right iliac fossa masses [7-9]. Secondary appendiceal involvement has been reported in patients with hepatic and splenic disease [7], while other cases had coexisting hepatic cysts discovered before or during investigation [6,11,12]. Cross-sectional imaging occasionally suggested a hydatid lesion, but appendiceal hydatidosis also mimicked an appendiceal mucocele [10] or a tubo-ovarian mass [9].

Operative management ranged from appendectomy alone to cyst excision, right hemicolectomy, and combined operations for multiorgan disease. Albendazole was used in most contemporary reports, but dose, timing, and duration varied. The small number and heterogeneity of reports preclude reliable estimates of recurrence or the superiority of open versus laparoscopic surgery (Table 2).

**Table 2:** Summary of published cases of appendiceal hydatid disease identified through the literature review and comparison with the present case. Summary of verified published cases of appendiceal hydatid disease identified through the literature review, including patient demographics, clinical presentation, disease pattern, preoperative diagnosis, treatment, and key clinical outcomes. The present case is included for comparison. Only reports with verifiable bibliographic records and sufficient clinical information were included. CT: computed tomography; CECT: contrast-enhanced computed tomography; IHA: indirect hemagglutination assay

Report	Patient	Presentation	Disease pattern	Preoperative diagnosis	Treatment	Outcome/learning point
Hajizadeh et al., 2013 [5]	58-year-old man	Anorexia, low-grade fever, mild abdominal pain	Hydatid elements in appendix	Not recognized; histological diagnosis	Appendectomy; mebendazole reported, schedule incompletely described	Serology negative at 3 months; symptom-free at 1 year
Mondal and Mandal, 2017 [6]	18-year-old woman	Acute appendicitis	Brood capsule in appendiceal lumen plus hepatic cyst	Ultrasound identified hepatic cyst but not appendiceal hydatidosis	Appendectomy with subsequent management of hepatic disease	Shows appendiceal involvement may coexist with silent hepatic disease
Bolandparvaz et al., 2010 [7]	47-year-old man	Six months of abdominal pain, fatigue, and anorexia	Secondary liver, appendix, and spleen involvement after previous hepatic surgery	CT and positive serology	Cystectomy, appendectomy, splenectomy; albendazole for 6 months	Illustrates secondary appendiceal involvement
De, 2009 [8]	56-year-old woman	Right iliac fossa mass with pain, nausea, vomiting, and fever	Primary right iliac fossa cyst presenting as an appendiceal mass	Ultrasound suggested hydatid cyst	Excision and appendectomy; albendazole 800 mg/day for 1 month	Well with normal ultrasound at 1 year
Agarwal et al., 2013 [9]	Adult woman	Pelvic/tubo-ovarian mass presentation	Hydatid cyst of the appendix with daughter cysts	Mimicked a tubo-ovarian mass	Surgical excision with appendiceal involvement managed	Highlights gynecological mimicry
Chaouch et al., 2019 [10]	75-year-old woman	Continuous right lower-quadrant pain	Infected calcified appendiceal hydatid cyst	CT suggested a complicated appendiceal mucocele	Open debulking surgery and right hemicolectomy	Histopathology established the diagnosis
Vera-Portilla et al., 2020 [11]	52-year-old man	Typical acute appendicitis	Appendiceal lesion with additional hepatic and peritoneal cysts	Diagnosed intraoperatively/postoperatively	Surgical management of appendiceal and intra-abdominal disease	Demonstrates disseminated intra-abdominal disease involving the appendix
Taghva Nakhjiri et al., 2024 [12]	5-year-old boy	One year of abdominal pain, anorexia, hepatomegaly, and pelvic mass	Cyst arising from appendiceal wall plus two hepatic cysts	Ultrasound/CT and positive IHA established hydatidosis	Resection with hypertonic-saline precautions; albendazole for 6 months	No recurrence reported; appendiceal origin recognized intraoperatively
Present case	43-year-old man	Acute appendicitis with systemic inflammatory response	Primary appendiceal lesion near base with intraoperative rupture	Ultrasound suggested fecalith; hydatid disease not recognized	Open appendectomy; hypertonic saline irrigation; albendazole in three cycles	CECT abdomen at three months showed no residual or additional hydatid cysts; no clinical recurrence was identified during 12 months of follow-up

Clinical spectrum and diagnostic challenge

The reviewed cases show that appendiceal hydatid disease is not a single clinicopathological entity. It may represent primary disease confined to the appendix, a cyst in the mesoappendix or adjacent peritoneum, intraluminal hydatid elements, or secondary involvement in multiorgan echinococcosis. This distinction matters because “primary” disease should be diagnosed only after common sites and other intra-abdominal locations have been assessed. In the present patient, follow-up CT showed no additional cysts, supporting primary appendiceal involvement.

Acute appendicitis remains the most deceptive presentation. Inflammatory markers, a non-compressible enlarged appendix, and an apparent fecalith can all be present without revealing the parasitic cause. More characteristic findings such as daughter cysts, detached membranes, or concomitant hepatic disease may raise suspicion, but these were absent from the preoperative ultrasound in our patient. Serology can support the diagnosis, yet its sensitivity varies by cyst location, stage, and burden and cannot reliably exclude isolated ectopic disease [2,15].

Pathogenesis and histopathology

The route by which *Echinococcus* reaches the appendix is uncertain. Proposed mechanisms include hematogenous or lymphatic dissemination that bypasses the hepatic and pulmonary filters, direct peritoneal implantation, and secondary spread from disease elsewhere. Histopathology is therefore central when the diagnosis is unsuspected. Characteristic findings include an acellular laminated membrane, an inner germinal layer, and, in fertile cysts, brood capsules, protoscolices, or refractile hooklets [5,16]. In the present case, the laminated and germinal layers were diagnostic.

Operative risk and management of spillage

Rupture is the most important immediate hazard. Hydatid fluid can precipitate intraoperative anaphylaxis, and viable protoscolices may seed the peritoneal cavity [13,14]. When an unexpected cystic lesion is encountered, manipulation should be minimized, the field isolated with protective packs, controlled suction used, and the anesthetic team alerted. Scolicidal solutions have historically been employed, but the concentration, contact time, and risk of chemical injury must be considered. In this case, immediate irrigation with hypertonic saline was undertaken, and no anaphylactic reaction occurred.

The evidence is insufficient to establish that open surgery is universally superior to laparoscopy for appendiceal disease. The safest approach depends on preoperative recognition, cyst size and location, surgeon expertise, the ability to isolate the lesion, and the likelihood of spillage. Conversion or a wider resection may be necessary when the lesion is infected, densely adherent, or indistinguishable from a neoplasm, as illustrated by the right hemicolectomy reported by Chaouch et al. [10].

Antiparasitic therapy and follow-up

Contemporary guidance is primarily based on hepatic and pulmonary cystic echinococcosis rather than appendiceal disease. WHO guidance emphasizes stage- and organ-specific management and the avoidance of iatrogenic complications [3]. CDC clinical guidance lists albendazole 400 mg twice daily for adults, generally for 1-6 months, and notes that praziquantel may be useful in the event of operative spillage [4]. Extrapolation to appendiceal disease should be individualized with infectious-disease or parasitology input. Our patient received three 28-day courses of albendazole 400 mg twice daily separated by 14-day intervals, a regimen consistent with historical cyclical benzimidazole practice.

Albendazole requires monitoring for hepatotoxicity and bone-marrow suppression. Imaging of the liver, lungs, and peritoneal cavity is important to define whether disease is primary or secondary and to detect recurrence after rupture. Serology may be used as an adjunct but should not replace imaging. Because secondary peritoneal cysts may take time to become visible, long-term follow-up is prudent even when an early CT is negative.

Relevance to gastroenterology

Although the definitive treatment in this case was surgical, the condition lies at the interface of gastroenterology, hepatology, infectious diseases, radiology, pathology, and surgery. Gastroenterologists may encounter hydatid disease as an incidental hepatic lesion, unexplained abdominal cyst, right iliac fossa mass, or postoperative referral after histological diagnosis. Recognition of atypical gastrointestinal manifestations, interpretation of hepatic and peritoneal imaging, coordination of antiparasitic therapy, and longitudinal surveillance are therefore directly relevant to gastroenterology practice.

Strengths and limitations

The main strength of this manuscript is the integration of a histologically confirmed case with a source-verified narrative synthesis that distinguishes appendiceal, secondary, and periappendiceal disease. Limitations include the rarity of the condition, reliance on isolated case reports, inconsistent reporting of drug regimens and follow-up, language and accessibility restrictions, and the absence of a systematic multidatabase search. A further limitation is that the original ultrasonographic images were not available to the authors; consequently, the ultrasonographic findings were derived from the contemporaneous radiology documentation rather than independently re-reviewed images. The review should therefore be interpreted as a clinically focused narrative synthesis rather than an estimate of incidence or treatment effectiveness.

## Conclusions

Appendiceal hydatid disease is an exceptionally rare cause or mimic of acute appendicitis. The available literature shows a broad spectrum from intraluminal parasitic elements to appendiceal-wall cysts, periappendiceal masses, and secondary multiorgan disease. Preoperative diagnosis is frequently missed. An unexpected cystic lesion near the appendix should be handled with minimal manipulation because rupture may cause anaphylaxis and peritoneal dissemination. Histopathology confirms the diagnosis, while postoperative staging, antiparasitic therapy, and prolonged imaging surveillance are essential. Framing the condition as a multidisciplinary gastrointestinal and parasitic disease, rather than solely a surgical curiosity, may improve recognition and continuity of care.
